# Impulsive Internet Game Play Is Associated With Increased Functional Connectivity Between the Default Mode and Salience Networks in Depressed Patients With Short Allele of Serotonin Transporter Gene

**DOI:** 10.3389/fpsyt.2018.00125

**Published:** 2018-04-10

**Authors:** Ji Sun Hong, Sun Mi Kim, Sujin Bae, Doug Hyun Han

**Affiliations:** ^1^Department of Psychiatry, Chung-Ang University Hospital, Seoul, South Korea; ^2^Industry Academic Cooperation Foundation, Chung-Ang University, Seoul, South Korea

**Keywords:** serotonin transporter gene, Internet gaming disorder, default mode network, salience network, major depressive disorder

## Abstract

Problematic Internet game play is often accompanied by major depressive disorder (MDD). Depression seems to be closely related to altered functional connectivity (FC) within (and between) the default mode network (DMN) and salience network. In addition, serotonergic neurotransmission may regulate the symptoms of depression, including impulsivity, potentially by modulating the DMN. We hypothesized that altered connectivity between the DMN and salience network could mediate an association between the 5HTTLPR genotype and impulsivity in patients with depression. A total of 54 participants with problematic Internet game play and MDD completed the research protocol. We genotyped for 5HTTLPR and assessed the DMN FC using resting-state functional magnetic resonance imaging. The severity of Internet game play, depressive symptoms, anxiety, attention and impulsivity, and behavioral inhibition and activation were assessed using the Young Internet Addiction Scale (YIAS), Beck Depressive Inventory, Beck Anxiety Inventory (BAI), Korean Attention Deficit Hyperactivity Disorder scale, and the Behavioral Inhibition and Activation Scales (BIS-BAS), respectively. The SS allele was associated with increased FC within the DMN, including the middle prefrontal cortex (MPFC) to the posterior cingulate cortex, and within the salience network, including the right supramarginal gyrus (SMG) to the right rostral prefrontal cortex (RPFC), right anterior insular (AInsular) to right SMG, anterior cingulate cortex (ACC) to left RPFC, and left AInsular to right RPFC, and between the DMN and salience network, including the MPFC to the ACC. In addition, the FC from the MPFC to ACC positively correlated with the BIS and YIAS scores in the SS allele group. The SS allele of 5HTTLPR might modulate the FC within and between the DMN and salience network, which may ultimately be a risk factor for impulsive Internet game play in patients with MDD.

## Introduction

Several national studies have demonstrated the relationship between impulsive Internet game play and major depressive disorder (MDD) (1–3). The severity of Internet use was associated with the risk of major depression in a group of 3,449 Korean middle school students (2). In a Hong Kong community (aged 18–60 years), the severity of Internet gaming disorder (IGD) was moderately strong correlation with the severity of depressive symptoms (3). A cross-sectional study of an Australian teenage community demonstrated that excessive Internet game play was associated with depression, anxiety, and poor health status (1). In studies on the treatment of patients with MDD and IGD, an improvement in depressive symptoms was associated with a reduction in the severity of IGD (4, 5). A comparison between the effects of bupropion and escitalopram on impulsive Internet game play in patients with MDD reported that decreased depressive symptoms were associated with an improvement of IGD in both groups (4). Furthermore, 12 weeks of bupropion treatment of 50 Patients with MDD and IGD also improved the symptoms of depression and IGD (5).

Recent studies suggested that IGD is caused by system level alterations between networks, rather than by the functional deficit within isolated regions (4–6). Many functional brain studies have demonstrated that human cognitive processes are orchestrated by a set of coherent spatiotemporal Independent Component networks (topographically organized human brain areas) (7). Our previous two studies demonstrated that the DMN and salience network were frequently associated in patients with MDD and IGD (4, 5). Sixty patients with MDD and IGD showed a failure to suppress DMN during an attentionally demanding task (the Wisconsin card sorting test) (5). In addition, decreased functional connectivity (FC) between the salience network and the DMN was associated with improved IGD symptoms and impulsivity in patients with MDD and IGD after 12 weeks of bupropion treatment (4). The FC in patients with MDD is thought to decrease between anterior DMN and posterior DMN, and increase between the salience network and anterior DMN (8). The salience network, which consists of the frontoinsular cortex, anterior cingulate, amygdala, and temporal pole, has been implicated in switching between the DMN and executive network (9). Grodin et al. reported that decreased volume within the salience network, including in the anterior insular (AInsular) and anterior cingulate, was negatively correlated with self-report impulsivity, decisional impulsivity, and compulsive measures (10).

Several neuroimaging studies have suggested that the serotonin transporter polymorphic region (5HTTLPR) plays an important neuromodulatory role on the DMN. In a positron emission tomography study, Hahn et al. (11) demonstrated that the density of the serotonin 1A (5-HT_1A_) receptor was associated with the FC within DMN. David et al. (12) suggested that the 5-HT_1A_ receptor density could be modulated by genetic variants of 5HTTLPR. In a study of the effects of 5HTTLPR variants on impulsivity in patients with MDD, Cha et al. suggested that the short allele of 5HTTLPR increases impulsivity by decreasing the FC between the DMN and superior frontal gyrus (SFG) (13). The 5HTTLPR in SLC6A4 was reported to lower the transcription of the gene encoding serotonin (14). Due to the regulation of serotonergic neurotransmission, the short allele of 5HTTLPR is thought to play a role in the symptoms including depressive mood, impulsivity, and neuroticism of patients with MDD (15). Furthermore, escitalopram and venlafaxine was reportedly less effective on patients with MDD, who had the short allele of 5HTTLPR, than on those with the long allele. In a study of venlafaxine treatment study, short allele of 5HTTLPR was less effective than long allele of 5HTTLPR in patients with MDD (16) In our previous study, Internet use in 166 high school students was more excessive in students who were homozygous for the short allelic variant of the serotonin transporter gene (ss-5HTTLPR), compared to healthy participants (17).

Therefore, we hypothesized that the 5HTLPR short allele was associated with increased FC within the DMN and salience network, as well as increased FC between the two networks, which may lead to impulsive Internet game play in patients with MDD.

## Materials and Methods

### Participants

A total of 60 patients with problematic Internet game play and MDD agreed to participate in the current research. All patients were diagnosed as MDD based on the Diagnostic Statistical Manual of Mental Disorder-V (DSM-V) (18). The criterion used to define IGD in the present study was the same as that used in our prior study (19). The criteria was as follows: (1) Internet game play time more than 4 h per day or 30 h per week, (2) Young Internet Addiction Scale (YIAS) score >50, (3) irritable, anxious, and aggressive behaviors upon request to stop Internet game play, (4) impaired behavior or distress, economic problems, and maladaptive life pattern as a result of problematic Internet game play, (5) disruptive diurnal rhythms (difficulty waking up during daytime hours due to reduced sleep at night related to Internet game play), and (6) loss of job or school truancy. The inclusion criteria were as follows: (1) diagnosed as MDD, (2) problematic Internet game play, (3) drug-naive, (4) over 18 years old, and (5) right-handed. The exclusion criteria included the following: (1) history or current episode of other psychiatric disorders, (2) IQ <80, (3) substance abuse history (except for alcohol and tobacco), (4) neurological or medical disorder, and (5) contraindication for magnetic resonance imaging (MRI) scanning. Of the 60 patients with MDD and IGD, 2 patients had low IQ (<80), 3 patients had a history of psychiatric medication, and 1 patient had a history of bipolar disorder. A total of 54 patients with MDD and IGD completed the final research protocol. The research protocol was approved by the Institutional Review Board of Chung Ang University Hospital. Written informed consent was provided by patients.

### Clinical Scale

The severity of Internet game play was assessed using the YIAS. The YIAS is a self-reporting scale for the severity of Internet use, with an internal consistency ranging from 0.90 to 0.91 (20). Depressive symptoms and anxiety were assessed using the Beck Depressive Inventory (BDI), with an internal consistency from 0.75 to 0.85 (21), and the Beck Anxiety Inventory (BAI), with Cronbach’s α = 0.93 (22), respectively. Attention and impulsiveness were assessed with the Korean Attention Deficit Hyperactivity Disorder scale (K-ARS) and Behavioral Inhibition and Activation Scales (BIS-BAS), respectively, which had an internal consistency from 0.77 to 0.89 (23) and 0.78 to 0.79 (24), respectively.

### MRI Acquisition and Preprocessing

Resting-state brain activity was assessed using 3T blood-oxygen-level dependent functional MRI (Philips Achieva 3.0 Tesla TX MRI scanner, TR = 3 s, 12-min scan, 240 volumes, 128 × 128 matrix, 40 slices at a 4.0-mm slice thickness). Preprocessing included despiking (AFNI: 3dDespike), motion correction (SPM 12b), coregistration to Magnetization Prepared RApid Gradient Echo image (SPM 12b), normalization, smoothing, temporal detrend (Matlab: detrend.m), bandpass filtering (Matlab: idealfilter.m), and voxelwise regression of identically bandpass filtered time series of six head motion parameters (realignment steps with six rigid-body parameters characterizing the estimated subject motion for each subject), degraded CSF, degraded white matter, and facial soft tissues (MATLAB), as previously described (25–27). All images were spatially normalized to the standard Montreal Neurological Institute space (SPM 12b), spatially smoothed with a 4-mm FWHM 3D Gaussian kernel to reduce spatial noise, linearly de-trended, and temporally filtered with a bandwidth of 0.01–0.08 Hz to reduce the effects of low-frequency drift and high-frequency noise, respectively. To address the possibility of micro-head movements affecting connectivity results (28, 29), time points with head motion >0.2 mm were censored, but no regression of the global signal was performed (30, 31). To assess the susceptibility of head motion, independent *t*-tests were performed to ensure that groups did not differ on rotation or translation parameter [translation: SS group = 0.039 ± 0.018, SL + LL group = 0.042 ± 0.016, *p* = 0.172; rotation: SS group = 0.0007 ± 0.00004, SL + LL group = 0.0008 ± 0.00003, *p* = 0.165]; average frame wise displacements were included as co-variates. The initial volumes (240 volumes) of each participant were used in the current study.

In group independent component analysis of the 54 participants in the current research, five brain circuits including the DMN, salience network, visual, dorsal attention network (DAT), and cerebellar network were best matched. Of the five regions, we selected two networks (DMN and salience). We extracted 11 regions of two brain networks [4 DMNs: middle prefrontal cortex (MPFC), right/left lateral parietal cortex, and posterior cingulate cortex (PCC); 7 salience networks: right/left AInsular, right/left supramarginal gyrus (SMG), right/left rostral prefrontal cortex (RPFC), and anterior cingulate cortex (ACC)] from rois toolbox folder (ver.15; www.Nitrc.org/projects/conn/rois). Fisher-transformed correlation coefficients were measured for each pair of regions of interest (ROI) in each participant. The FC was calculated between ROIs using the CONN-fMRI FC toolbox (ver.15; www.Nitrc.org/projects/conn). Between-group effects were considered significant with a cluster level false discovery rate (FDR; *q* < 0.05), considering the multiple comparison correction of 55 pairs of 11 regions.

### Genotyping

Genotyping was performed at Labgenomics, Korea. Genomic DNA was extracted from blood (stored frozen) using a G-DEX™ II Genomic DNA Extraction Kit (Intron Biotechnology, Korea), according to the manufacturer’s protocol. The region encompassing 5HTTLPR polymorphisms was amplified with the primers FORWARD: 5′-GGCGTTGCCGCTCTGAATGC-3′ and REVERSE: 5′- GAGGGGACTGAGCTGGACAACCAC-3′ *via* a polymerase chain reaction in 2.5 mM 7-deaza dNTP mix (Roche, Germany). Amplicons were resolved on a 2% agarose gel (Solgent, Korea) and visualized under a UV transilluminator. Herein the 528- and 484-bp bands will be called the L and S alleles of 5HTTLPR, respectively.

### Statistical Analyses

An independent *t-*test was performed to compare the mean differences of age, school years, BDI, BAI, K-ARS, YIAS, BAS, and BIS scores between the SS allele group and SL + LL allele group. Controlling for age, an ANCOVA was applied to measure differences in FC between the SS allele and SL + LL allele groups. Controlling for age, YIAS score, and BAS score, partial correlation was performed to assess the association between clinical scales, as well as between clinical scales and brain connectivity.

## Results

### Demographic and Clinical Characteristics

There were no significant differences in demographic data between SS allele group and SL + LL allele group, but impulsivity and severity of IGD were higher in SS allele group than those observed in SL + LL allele group. The sample in the current study consisted of 54 patients with MDD (all men) with a mean age of 21.7 ± 3.6 years (range: 18–28 years). The distribution of the current sample was as follows: SS allele (*n* = 28), SL allele (*n* = 21), and LL allele (*n* = 5). The current sample satisfied the Hardy–Weinberg equilibrium (χ^2^ = 0.13, df = 1, *p* = 0.71). There were no significant differences in age, school years, BDI, BAI, K-ARS, and BIS scores between the SS allele group and SL + LL allele group. However, the SS allele group had higher YIAS and BAS scores than SL + LL allele group (Table 1).

**Table 1:** Demographic and clinical characteristics.

	SS-allele group (28)	SL + LL allele group (26)	Statistics
Age	22.3 ± 7.6	20.0 ± 4.6	*t* = 1.56, *p* = 0.12
School years	11.7 ± 1.8	11.8 ± 1.6	*t* = 0.11, *p* = 0.91
BDI	22.3 ± 7.6	20.8 ± 4.5	*t* = 1.41, *p* = 0.16
BAI	12.2 ± 6.1	11.5 ± 7.0	*t* = 0.40, *p* = 0.69
K-ARS	12.1 ± 6.2	11.6 ± 5.3	*t* = 0.31, *p* = 0.75
YIAS*	65.8 ± 11.1	60.0 ± 7.7	*t* = 2.31, *p* = 0.03
BAS*	23.0 ± 3.2	19.8 ± 3.2	*t* = 3.01, *p* < 0.01
BIS	28.3 ± 8.5	26.2 ± 8.5	*t* = 0.87, *p* = 0.38

*BDI, Beck Depressive Inventory; BAI, Beck Anxiety Inventory; K-ARS, Korea Attention Deficit Hyperactivity Disorder Scale; YIAS, Young Internet Addiction Scale; BIS-BAS, Behavioral Inhibition and Activation Scales*.

*Statistically significant.

### Comparing Brain FC of the DMN and Salience Network Between the SS and SL + LL Allele Groups

**Figure 1:**
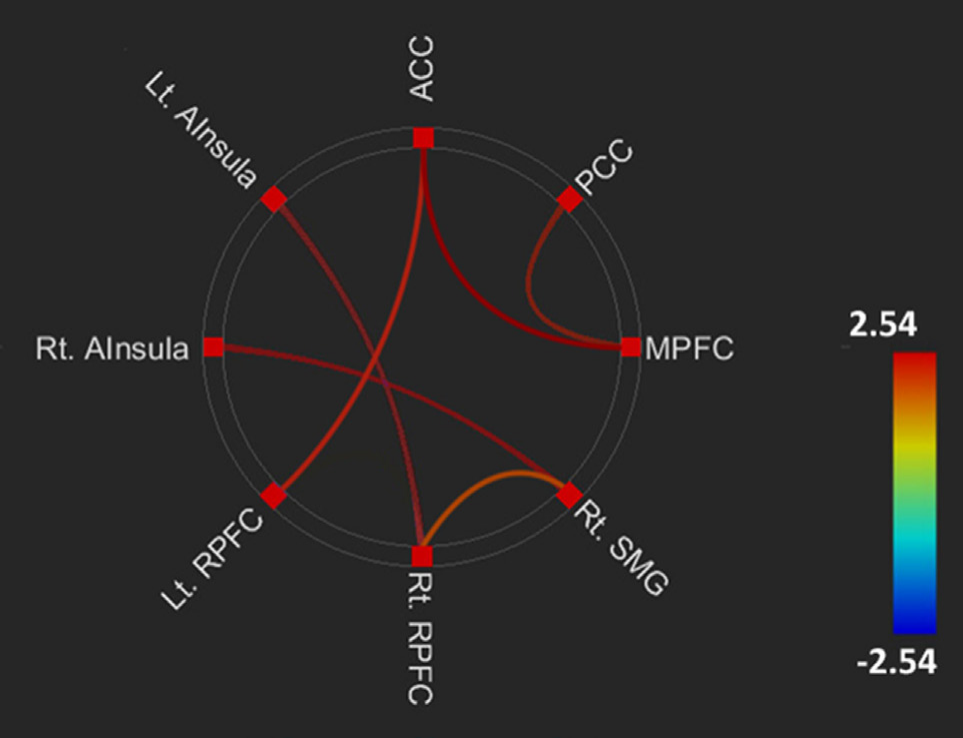
Comparing brain functional connectivity of brain areas between the SS and SL + LL allele groups. Default mode network.middle prefrontal cortex (DMN.MPFC) to posterior cingulate cortex (DMN.PCC): *t* = 2.42, FDRq = 0.02; left salience network.anterior insular (SAL.AInsula) to right rostral prefrontal cortex (SAL.RPFC): *t* = 2.42, FDRq = 0.02; right SAL.AInsula (SAL.AInsula) to right SAL supramarginal gyrus (SAL.SMG): *t* = 2.40, FDRq = 0.02; right SAL.SMG to right SAL.RPFC: *t* = 2.02, FDRq < 0.05; SAL.ACC to left SAL.RPFC: *t* = 2.15, *p* = 0.04; DMN.MPFC to anterior cingulate cortex (SAL.ACC): *t* = 2.61, FDRq = 0.01.

The FC within DMN and salience network, and between the networks, in the SS allele group (serotonin deficit) was higher than that in the SL + LL allele group. Compared to the SL + LL allele group, the SS allele group had greater FC within the DMN, including MPFC to PCC (*t* = 2.42, *p* = 0.02), and the salience network, including the right SMG to right RPFC (*t* = 2.02, *p* < 0.05), right AInsular to right SMG (*t* = 2.40, *p* = 0.02), ACC to left RPFC (*t* = 2.15, *p* = 0.04), and left AInsular to right RPFC (*t* = 2.42, *p* = 0.02), and between the DMN and salience network, including the MPFC to ACC (*t* = 2.61, *p* = 0.01) (Figure 1).

### Correlation Between Clinical Scales and Brain Connectivity

Impulsivity and severity of IGD were associated with the FC between DMN and salience network in patients with MDD and IGD in the SS allele group alone.

**Figure 2:**
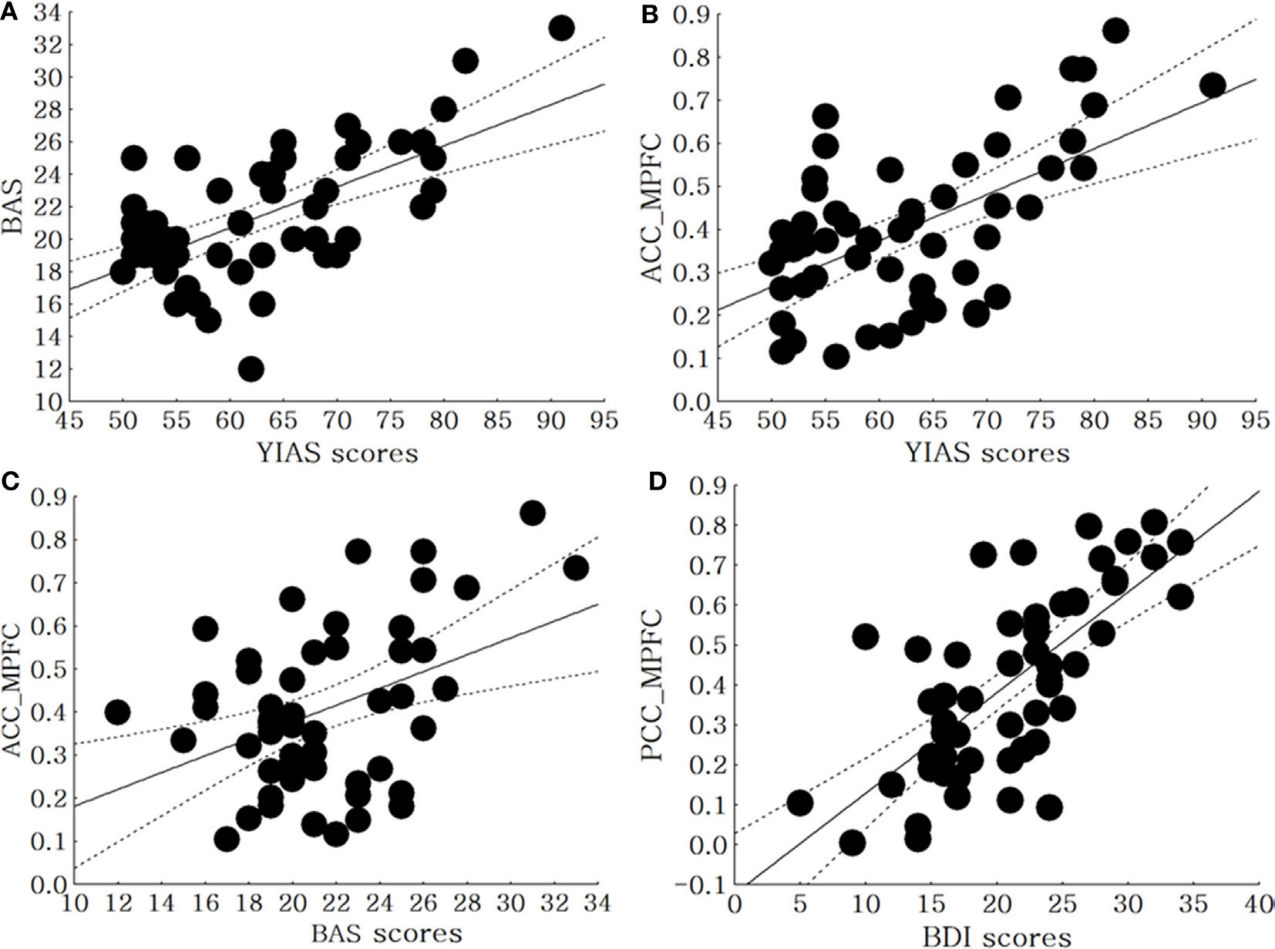
Correlation between clinical scales and brain connectivity in all patients with major depressive disorder (MDD) and Internet gaming disorder (IGD). **(A)** Correlation between the BAS scores and the Young Internet Addiction Scale (YIAS) scores in all patients with MDD with IGD (*r* = 0.63, *p* < 0.01), **(B)** correlation between the YIAS scores and FC between anterior cingulate cortex (ACC) to middle prefrontal cortex (MPFC) in all Patients with MDD with IGD (*r* = 0.58, *p* < 0.01), **(C)** correlation between the BAS scores and FC between ACC to MPFC in all patients with MDD with IGD (*r* = 0.42, *p* < 0.01), **(D)** correlation between the Beck Depressive Inventory (BDI) scores and FC between PCC to MPFC in all patients with MDD with IGD (*r* = 0.51, *p* < 0.01).

In all patients with MDD and IGD, there was positive correlation between the BAS score and YIAS score (*r* = 0.63, *p* < 0.01). The BDI scores had no significant correlations with YIAS scores (*r* = 0.03, *p* = 0.79) and BAS scores (*r* = 0.07, *p* = 0.61). The YIAS scores (*r* = 0.58, *p* < 0.01) and BAS scores (*r* = 0.42, *p* < 0.01) were positively correlated with FC from the ACC to MPFC. Furthermore, the BDI score was positively correlated with the FC from the PCC to the MPFC (*r* = 0.71 *p* < 0.01) (Figure 2). The BDI scores were not correlated with the FC from the ACC to the MPFC (*r* = 0.11, *p* = 0.49). The YIAS scores (*r* = 0.28, *p* = 0.06) and BAS scores (*r* = 0.25, *p* = 0.08) were not correlated with the FC from the PCC to the MPFC.

**Figure 3:**
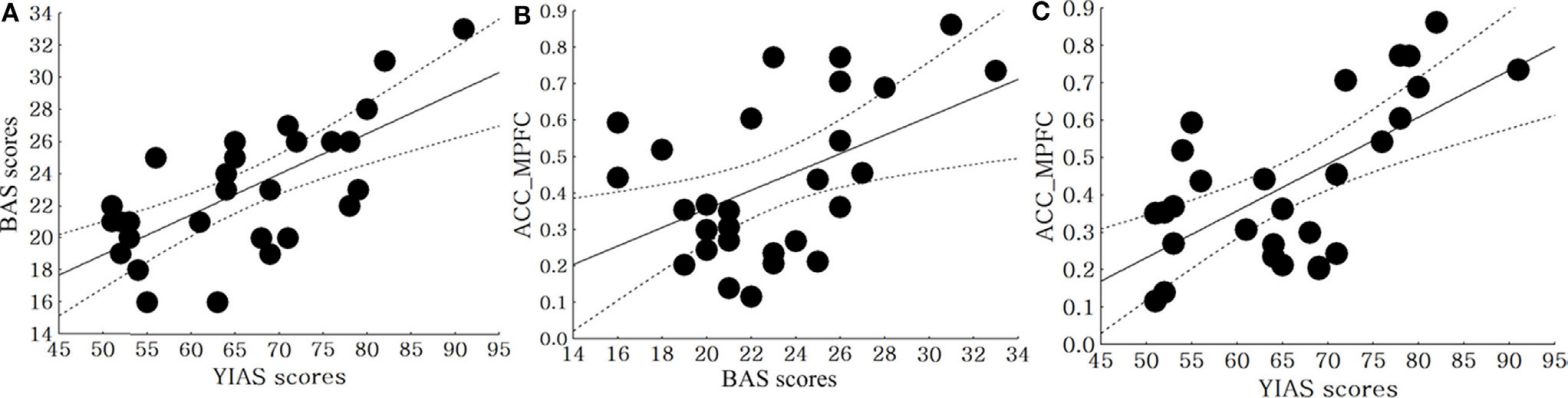
Correlation between clinical scales and brain connectivity in the SS allele and SL + LL allele groups. **(A)** Correlation between the BAS and Young Internet Addiction Scale (YIAS) scores in the SS allele group (*r* = 0.68, *p* < 0.01), **(B)** correlation between the BIS scores and FC from anterior cingulate cortex (ACC) to middle prefrontal cortex (MPFC) in SS allele group (*r* = 0.48, *p* = 0.01), **(C)** correlation between the YIAS scores and FC from ACC to MPFC in SS allele group (*r* = 0.64, *p* < 0.01).

In the SS allele group, there was a positive correlation between the BAS score and YIAS score (*r* = 0.68, *p* < 0.01). Even after controlling for each of the two measures, the BAS score (*r* = 0.48, *p* = 0.01) and YIAS score (*r* = 0.64, *p* < 0.01) were positively correlated with the FC from the ACC to the MPFC (Figure 3). In the SL + LL allele group, there was no correlation between BAS score and YIAS score (*r* = 0.34, *p* = 0.09). After controlling for each of the two measures, the BAS scores (*r* = 0.21, *p* = 0.32) and YIAS score (*r* = 0.29, *p* = 0.06) were not correlated with the FC from the ACC to the MPFC.

## Discussion

### Comparison of Clinical Characteristics Between the SS and SL + LL Allele Groups

In the current study, there were no significant differences in depressive symptoms between the SS and SL + LL allele groups. The effects of the 5HTTLPR polymorphism on depressive symptoms in patients with MDD are controversial. In pharmacological studies, the S allele is thought to predict a bad response to medication in diseases associated with serotonergic system dysfunction (32, 33). A meta-analysis demonstrated that the 5HTTLPR polymorphism did not play a major role in predicting the progress of mood symptoms in Asian patients with MDD (34).

However, we found that the SS allele group had higher YIAS and BAS scores than the SL + LL allele group. In our previous study of 166 adolescent, adolescents with excessive Internet use had higher frequencies of the SS allele of 5HTTLPR and harm avoidance, compared to those in the SL + LL allele group (17). In addition, there was a positive correlation between BAS scores and YIAS scores. An association between impulsiveness and the 5HTTLP polymorphism has also been reported in other studies. When the effect of fluoxetine on reducing impulsiveness and irritability was assessed using the Modified Overt Aggression Scale, patients with L carrier (SL + LL alleles) borderline personality disorder showed a better response than S carriers (35). Taken together, we cautiously suggest that short allele of 5HTTLPR is associated with impulsive Internet game play in patients with MDD and IGD.

### Comparing Brain Functional Connectivity of the DMN and Salience Network Between the SS and SL + LL Allele Groups

All patients with MDD and IGD in the present study showed a positive correlation between the BDI scores and FC within the DMN. Mulders et al. reported the increase in the FC within DMN and within salience network in patients with MDD, compared to healthy control participants (8). In addition, the FC between DMN and salience network was higher in patients with MDD than in healthy participants (8). Moreover, IGD symptoms in patients with MDD were reportedly associated with increased FC between the DMN and salience network (4, 5).

Considering the increased FC within (and between) the DMN and salience network in the SS allele group, our genetic neuroimaging findings suggest that the serotonergic system may play a role in impulsive Internet game play in patients with MDD. Previous reports already demonstrated that the deficit of serotonin neurotransmission in the DMN and salience network were associated with the severity of mood symptoms, chemical addictive symptoms, and impulsive Internet gaming symptoms (13, 36). Patients with MDD, who have the S allele, show microstructural white matter abnormalities within the frontolimbic networks and a lower remission rate, compared to patients with MDD who have the LL allele (36). Furthermore, Cha et al. reported that the S allele genotypes of 5HTTLPR (SS and SL) were associated with lower FC between the posterior DMN and SFG (13). In that study, path modeling analysis demonstrated that increased FC between the DMN and SFG would mediate impulsivity in patients with MDD (13). Increased FC within the DMN and salience network was also found in codeine-dependent patients; the FC within those areas was associated with impulsivity (37). Increased FC between the DMN and salience network was also reported in patients with IGD who had a childhood history of ADHD (38).

In our results, the SS allele group showed greater FC within the DMN and salience network, and between these networks, compared to the SL + LL allele group. In addition, BAS scores and YIAS scores were positively correlated with the FC between the DMN and salience network in SS allele group alone. Taken together, our results suggest that the short allele of 5HTTLPR may increase FC within the DMN and salience network, which may subsequently aggravate impulsive Internet game play in patients with MDD.

### Limitations

A couple of limitations in the current study must be noted. First, the relatively small number of participants prevented the generalization of the current results. Second, there were no neurocognitive tests for assessing the function of the DMN or salience network. Thus, future studies should consider assessing a larger cohort of participants and applying a neurocognitive test.

## Conclusion

The current results suggested that the SS allele of 5HTTLPR can be a risk factor for impulsiveness and excessive Internet game play in patients with MDD and IGD. In addition, the SS allele of 5HTTLPR may modulate FC, not only within the DMN and salience network but also between the networks.

## Ethics Statement

The research protocol was approved by the Institutional Review Board of Chung Ang University Hospital. The study was conducted in accordance with the ethical standards of the Helsinki Declaration of 1964 and subsequent amendments or similar ethical standards. Written informed consent was obtained from all participants.

## Author Contributions

JH, SK, and DH contributed to patient recruitment, and data collection and processing. JH, SB, and DH analyzed the data. All authors participated to drawing up the manuscript and were involved in the intellectual workup for the article. All authors read and approved the final manuscript.

## Conflict of Interest Statement

The authors declare that the research was conducted in the absence of any commercial or financial relationships that could be construed as a potential conflict of interest.
